# Multilayer Methacrylate-Based Wound Dressing as a Therapeutic Tool for Targeted Pain Relief

**DOI:** 10.3390/ma16062361

**Published:** 2023-03-15

**Authors:** Tanja Zidarič, Kristijan Skok, Kristjan Orthaber, Matevž Pristovnik, Lidija Gradišnik, Tina Maver, Uroš Maver

**Affiliations:** 1 Institute of Biomedical Sciences, Faculty of Medicine, University of Maribor, Taborska ulica 8, 2000 Maribor, Slovenia; 2 Department of Pathology, Hospital Graz II, Location West, Göstinger Straße 22, 8020 Graz, Austria; 3 Department of Pharmacology, Faculty of Medicine, University of Maribor, Taborska ulica 8, 2000 Maribor, Slovenia

**Keywords:** wound dressings, pain relief, superparamagnetic nanoparticles, methacrylate

## Abstract

This study presents an innovative wound dressing system that offers a highly effective therapeutic solution for treating painful wounds. By incorporating the widely used non-steroidal anti-inflammatory drug diclofenac, we have created an active wound dressing that can provide targeted pain relief with ease. The drug was embedded within a biocompatible matrix composed of polyhydroxyethyl methacrylate and polyhydroxypropyl methacrylate. The multilayer structure of the dressing, which allows for sustained drug release and an exact application, was achieved through the layer-by-layer coating technique and the inclusion of superparamagnetic iron platinum nanoparticles. The multilayered dressings’ physicochemical, structural, and morphological properties were characterised using various methods. The synergistic effect of the incorporated drug molecules and superparamagnetic nanoparticles on the surface roughness and release kinetics resulted in controlled drug release. In addition, the proposed multilayer wound dressings were found to be biocompatible with human skin fibroblasts. Our findings suggest that the developed wound dressing system can contribute to tailored therapeutic strategies for local pain relief.

## 1. Introduction

The critical and complicated process of wound healing and the complexity of the skin as an organ [1] are the main reasons why skin wounds and skin-associated diseases are particularly challenging for physicians worldwide [2,3,4]. We are currently dealing with an overwhelming number of different wound dressings, which is, among other things, evidence that we still lack a comprehensive understanding of wound care and management [4]. The characteristics of an ideal wound dressing are diverse and depend on the type of wound. However, common factors include maintaining a moist environment, stopping blood loss, removing excess exudate, providing thermal insulation and mechanical protection, and acting as a barrier to microorganisms [4,5,6,7,8]. Current research focuses on advanced therapeutic wound dressings actively involved in wound healing, which requires a multidisciplinary approach [4,7,9,10,11]. The composition of the exudate (pH, ionic strength, presence of various growth and inflammatory factors, etc.) differs in different wound types, which can be used to adapt the drug release profiles to the specifics of the wound type. Advances in materials science and technology, as well as skin tissue engineering, have motivated the development and construction of innovative dressing materials capable of releasing drugs to prevent infection and promote healing [12,13] or even detecting infection conditions in the wound environment [14,15,16,17,18].

Incorporating active ingredients highly depends on the materials used [11]. The demand for cheap raw materials led to the establishment of synthetic polymeric composites available in various forms, for example, foams, thin films, membranes, and meshes [19]. Synthetic polymers, such as polyhydroxyethyl methacrylate (PHEMA) and polyhydroxypropyl methacrylate (PHPMA), have attracted considerable attention as biocompatible materials for biomedical applications. These polymers are characterised by excellent water absorption and retention under various conditions. As hydrogels, they are widely used to fabricate contact lenses, implants, tissue scaffolds, and drug delivery systems [20]. As a class of synthetic hydrogels, they offer greater latitude for designing and controlling material properties. Because these polymers do not contain animal proteins, the potential for allergic reactions is also lower. Although PHEMA and PHPMA lack the natural biological activity of natural polymers such as collagen, which can be important in promoting cell adhesion and proliferation, their surface can be easily modified or charged to promote cell attachment or differentiation [21,22,23]. In addition, they also have a cooling and soothing effect, which is desirable in the pain management of skin injuries [8,24,25,26].

Spin coating is one of the most commonly used processes for preparing thin film coatings for biomedical applications. It allows the production of well-organised mono- and multilayer films over large areas with smooth and homogeneous surfaces. In addition, it can be performed under ambient conditions, making it one of the most effective and easiest methods for developing novel coatings for medical implants, medical devices, and wound dressings [7,25,27,28,29]. Multilayer and multifunctional wound dressings, especially those manufactured using the layer-by-layer (LbL) technique, have been available for comprehensive wound care for some time. However, the existing products often fail in the challenging aspects of wound treatment, such as controlled therapeutic effects or wound-type-specific healing [7,30]. LbL self-assembly technology has been shown to be effective in developing innovative drug delivery systems, especially when controlling drug release on the mechanistic level [31,32,33,34]. Of particular interest are novel wound dressings with embedded nanoparticles (NPs), which represent the “next generation” of drug delivery systems for multiple modes of administration, including intravenous, transdermal, or topical delivery [7,33,35,36].

For numerous biomedical applications, it is advantageous to integrate superparamagnetic nanoparticles (SMNPs) into a biocompatible material for targeted delivery and retention [20,37]. NPs have generally gained popularity as nanocarriers for topical, transdermal, and intravenous drug delivery systems [7,35,36,38,39]. They have been shown to enhance the penetration of large molecules across the stratum corneum without compromising the skin barrier to pathogenic microbes [40,41,42,43]. Another possible application is targeted drug delivery due to their special physical, chemical, and optical properties. The latter properties allow a non-invasive delivery of drugs in the target tissue and thermal or photo-guided release [44,45,46]. SMNPs such as FePt nanoparticles (FePt NPs) [20,41,47,48] are of particular interest to our study because, as the name implies, they might allow controlled magnetic treatment, retention, and manipulation [49]. The coupling of SMNPs and polymeric matrices can lead to a remarkable refinement of the surface properties of SMNPs (e.g., FePt NPs) in terms of drug loading and effective drug distribution, whether for targeted delivery or sustained release. To maintain the SMNPs’ biocompatibility in the human body and prevent sedimentation in tissues, they should be coated with biocompatible polymers [45,48,49,50,51,52,53]. At the same time, they also enable tailoring of the polymer matrix properties [20].

In this study, the spin coating technique was used to develop a multilayered wound dressing system for sustained release of diclofenac (DCF). The latter was hypothesised to be possible through the combination of polyhydroxyethyl methacrylate (PHEMA), polyhydroxypropyl methacrylate (PHPMA), sodium deoxycholate (NaDOC), and active ingredients (i.e., superparamagnetic FePt NPs and DCF, as a pain-relieving drug) in an LbL manner. DCF is a non-steroidal anti-inflammatory drug (NSAID) that provides long-lasting pain relief. It is approved for topical analgesia in the form of sodium salts. Although NSAIDs are usually administered to help the healing process of acute wounds, there is evidence that when they act locally, they can help chronic wounds overcome the inflammatory phase by relieving excessive inflammation [10,54,55,56]. Furthermore, DCF is a standard treatment for actinic keratosis [57,58,59,60,61]. The wound dressing formulation we developed would provide a proven analgesic effect due to sustained pain relief, achieved by the tailored drug release properties of the prepared material. The developed material combines the intrinsic permeability of SMNPs into human tissues [46] to facilitate the penetration and sustained release of the incorporated drug and improve the physicochemical properties of the polymer matrix [62]. All the prepared samples were characterised by their physicochemical properties, biocompatibility, and cytotoxicity. In addition, drug release was evaluated using an in vitro assay, and safety of the formulation was studied on human skin fibroblasts.

## 2. Materials and Methods

### 2.1. Materials

Polyhydroxyethyl methacrylate (PHEMA; Mw: 515 kDa, CAS num.: 9003-42-3), polyhydroxypropyl methacrylate (PHPMA; Mw: 30–50 kDa, CAS num.: 21442-01-3), diclofenac sodium salt (NaDOC; Mw: 318.13, CAS num.: 15307-79-6), sulphuric acid (H_2_SO_4_; Mw: 98.08, CAS num.: 7664-93-9), and hydrogen peroxide (H_2_O_2_; Mw: 34.01, CAS num.: 7722-84-1) were purchased from Sigma-Aldrich (Darmstadt, Germany), while absolute ethanol (EtOH, Mw: 46.07, CAS num.: 64-17-5) was purchased from the French subsidiary of Sigma-Aldrich (Lyon, France). DCF, an NSAID, was also purchased from Sigma-Aldrich (Lyon, France). The superparamagnetic iron-platinum nanoparticles (FePt NPs; average diameters of 6 ± 1 nm), developed under the Slovenian Research Agency-funded project J2-6760, were kindly provided by Ass. prof. Kristina Žužek Rožman (Institute “Jožef Stefan”, Department of Nanostructured Materials, Ljubljana, Slovenia [20]. All materials were used as supplied without further modification before sample preparation or testing. Ultrapure water (18.2 MΩ cm at 25 °C) prepared using an ELGA Purelab water purification system (Veolia Water Technologies, High Wycombe, UK) was used for all water-based solutions.

Atomic flat silicon wafers (Si-wafers, Topsil, Winsen, Germany) were used as substrates for the LbL spin coating of multilayer dressings.

Human-derived skin fibroblasts (ATCC-CCL-110, Detroit 551) were purchased from LGC Standard (Bury, Lancashire, UK) and used for biocompatibility testing of the fabricated samples. Dulbecco’s Modified Eagle Medium (ADMEM), Advanced Dulbecco’s Modified Eagle Medium/Nutrient Mixture F-12 (ADMEM/F-12), and fetal bovine serum (FBS) were purchased from Thermo Fisher Scientific (Schwerte, Germany). Phosphate-buffered saline (PBS), L-glutamine, penicillin G sodium salt, streptomycin sulphate salt, and MTT tetrazolium salt (3-(4,5-dimethylthiazol-2-yl)-2,5-diphenyltetrazolium bromide) were supplied by Sigma-Aldrich (Darmstadt, Germany).

#### 2.1.1. Preparation of Solutions

Four different formulations were developed for preparing the multilayer dressings. Formulation 1 (F1: PHEMA/PHPMA/NaDOC) was the “base polymer” solution of PHEMA (10 mg/mL, dissolved in EtOH), PHPMA (1 mg/mL, dissolved in ultrapure water), and NaDOC (10 mg/mL, dissolved in ultrapure water) mixed in a ratio of 1:0.1:1. Formulation 2 (F2: PHEMA/PHPMA/NaDOC/DCF) was the drug-containing solution, consisting of DCF (10 mg/mL, dissolved in ultrapure water) added to PHEMA/PHPMA/NaDOC (prepared in the same way as F1) to obtain the final formulation in the ratio 1:0.1:1:1. Formulations 3 (F3) and 4 (F4) were FePt NPs containing suspensions with and without DCF, respectively [20]. For the preparation of F3 (PHEMA/PHPMA/NaDOC/FePt), 1 mL of an aqueous suspension of FePt NPs (1 mg/mL) was added to 2.5 mL solution of PHEMA/PHPMA/NaDOC (prepared as in F1). For F4 (PHEMA/PHPMA/NaDOC/FePt/DCF), PHEMA/PHPMA/NaDOC/FePt and the active ingredient drug DCF (1 wt.% in the final solution) were mixed in a ratio of 1:0.1:1:1:1. All solutions (summarised in Table 1) were freshly prepared before use.

#### 2.1.2. Substrate Preparation

Silicon wafers (Si-wafers) have been used as a base substrate for multilayer dressing fabrication due to their atomically flat surface and higher thermal conductivity, resulting in smaller thickness differences than glass or plastic [63,64]. Before use, Si-wafers were cut into 8 × 8 mm^2^ pieces and immersed in a “piranha solution” (98 wt.%H_2_SO_4_ and 30 wt.% H_2_O_2_ in 70:30 *v/v* ratio) for 15 min at room temperature, rinsed with ultra-pure water and dried in a stream of dry nitrogen of high purity (99.999% wt.%, Messer, Ruše, Slovenia).

#### 2.1.3. Preparation of Multilayer Dressings

**Figure 1:**
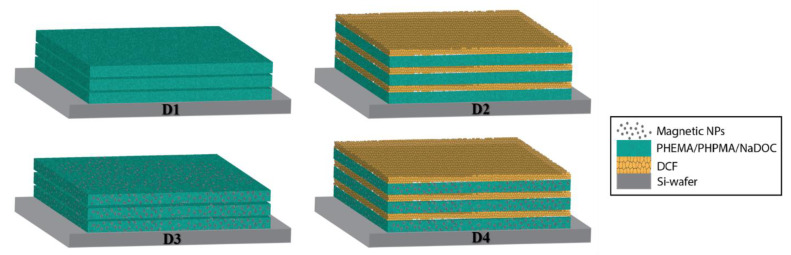
Graphical presentation of preparation of multilayer pain-relief dressings.

All four formulations mentioned in Section 2.1.1 were used to prepare multilayer dressings on the substrate. The multilayer dressings in an LbL configuration were created by alternating the spin-coating of different formulations. Using spin coating with a POLOS, SPIN 150i spin coater (SPS GmbH, Ingolstadt, Germany), 50 μL of respective formulation were deposited on the substrate. The LbL procedure was performed by applying the first formulation to the static substrate and spinning it at an initial speed of 1000 rpm with an acceleration of 1000 rpm/s to a final rotation of 2500 rpm for 80 s. The first formulation was then applied to the static substrate. After applying the first layer, the procedure was repeated in the same way with other formulations, as shown in Figure 1 and Table 2, where the graphical representation and the list of the model dressing types are presented.

### 2.2. Characterisation

#### 2.2.1. Contact Angle Measurement

The hydrophilicity of the samples was determined by the static water contact angle method (SCA) performed on an OCA 15 Pro system (Dataphysics, Filderstadt, Germany) at ambient temperature. All measurements were performed on three independent samples (for each sample on Si-wafers) on three different sample areas. For this purpose, one drop of ultrapure water (3 μL) was applied to each sample. Each SCA value averaged at least three water drops per surface. An average SCA value with the standard error was calculated using the hardware manufacturer’s software (version 20.2.0).

#### 2.2.2. Attenuated Total Reflectance Infrared Spectroscopy (AFT-IR)

ATR-IR spectra of the spin-coated samples were acquired using an Agilent Cary 630 FTIR spectrometer (Agilent, Santa Clara, CA, USA) equipped with a diamond crystal (ATR module, measurement range 400–650 cm^−1^). A total of 24 scans were performed on each sample surface for each spin-coated layer [59,65]. The IR permeability of the samples was evaluated using MicroLabPC 4.0 software (Agilent, Santa Clara, CA, USA) and plotted as absorbance (*y*-axis) versus wavenumber (*x*-axis), which was additionally processed using OriginPro 8.5 software (OriginLab, Stoke Mandeville, Buckinghamshire, UK).

#### 2.2.3. Atomic Force Microscopy

Atomic force microscopy (AFM) was used to characterise the spin-coated multilayer dressings’ topography and surface roughness. Before analysis with a Keysight 7500 AFM (Keysight Technologies, Santa Rosa, CA, USA), samples were mounted on round-shaped metal disc sample holders. Images were recorded after the samples were dried in a stream of dry, high-grade (99.999 wt.%) nitrogen gas to minimise possible interactions between the sample surface and silicon cantilevers (ATEC-NC-20, Nanosensors, Wetzlar, Germany, with a resonant frequency of 210–490 kHz and a force constant of 12–110 N m^−1^) due to adsorbed water. All measurements were performed at ambient temperature. For imaging, the samples were scanned with a scan size of 10 × 10 μm^2^ and a resolution of 2048 × 2048 pixels [65,66]. Gwyddion software (Czech Metrology Institute, Prague, Czech Republic) was used to process all images and calculate the corresponding roughness parameters.

### 2.3. Functional Testing

#### 2.3.1. In Vitro Drug Release Testing

The release of DCF from the multilayer dressings was studied using an Automated Transdermal Diffusion Cells Sampling System (Logan System 912-6, Somerset, NJ, USA) by determining the changes in the amounts of incorporated drug released (mass concentration (γ), mass (m), and percentage of drug released (%)) as a function of exposure time (t) [10,27,65,66]. All measurements of the release from the drug-containing (DCF) samples with and without FePt NPs, i.e., D2 and D4, were performed in phosphate buffer solution (PBS, pH = 7.4) at a constant release medium temperature of 37 °C. Stirring was constant at 50 rpm throughout the experiment. The D2 and D4 samples were placed in a Franz diffusion cell with the coated part exposed to PBS. The released DCF was sampled at different exposure times (1 min, 5 min, 10 min, 20 min, 30 min, 60 min, 120 min, 180 min, 240 min, 300 min, 360 min, and 1440 min) over 24 h. The withdrawn sample volume was replaced with PBS solution at 37.0 °C. The resulting dilutions enabled sink conditions throughout the experiment. These dilutions were considered in the release profile calculations using the Beer–Lambert law [59,65]. A UV-Vis spectrophotometer was used to determine the amounts of DCF released (Cary 60 UV-Visible Spectrophotometer, Agilent, Waldbronn, Germany) by quantifying the absorption band at 276 nm (characteristic of DCF). Three replicates were performed for each D2 and D4 sample, and an average concentration was reported with a standard error. Using UV-Vis spectrophotometry, the amount of DCF released was measured daily until no change in concentration occurred. This amount was used as the total incorporated drug amount for further calculations.

To evaluate the release profiles, the results of the in vitro release study were fitted with a modified Korsmeyer–Peppas model describing the release from polymer-based formulations [65,67]. For all curves shown, the confidence interval was determined as *±ts/√x*, where *t* is the Student’s *t*-distribution, *s* is the standard deviation, and *x* is the number of measurements [27,65].

#### 2.3.2. Cell Cultures and Viability Testing

The effect of all samples on the cell viability of human skin fibroblasts (ATCC-CCL -110, Detroit 551, LGC Standards, Teddington, UK) was studied by the reduction reaction of MTT tetrazolium salt (3-(4,5-dimethylthiazol-2-yl)-2,5-diphenyltetrazolium bromide) [68], which was purchased from Sigma Aldrich, Darmstadt, Germany. This is a common and reliable method for evaluating cell proliferation and growth. The assay is based on reducing yellow MTT by metabolically active cells, forming nicotinamide adenine dinucleotide and/or nicotinamide adenine dinucleotide phosphate. The reaction forms a purple intracellular formazan that can be dissolved and quantified by spectrophotometric methods [68,69]. In our case, the extract dilution method of the MTT assay was performed according to Mosmann [68]. All samples (8 × 8 mm^2^) were first sterilised with UV light for 30 min. After sterilisation, samples were soaked in 3 mL of Advanced Dulbecco’s modified Eagle Medium supplemented with 5 wt.% fetal bovine serum (ADMEM + 5 wt.% FBS) and incubated for 24 h at 37 °C in an atmosphere containing 5 wt.% CO_2_. Skin fibroblasts (10,000 cells/well) were seeded onto a 96-well microtiter plate (P96) with a final volume of 100 µL ADMEM + 5 wt.% FBS. Supernatants of the initial samples and their dilutions (1:2, 1:4, 1:8, and 1:16) were added to the cells in four parallel steps after 24 h incubation at 37 °C in an atmosphere containing 5 wt.% CO_2_. Pure cell growth medium (ADMEM + 5 wt% FBS) was used as a control. Cell viability (i.e., cytotoxicity) was determined after 24 h by measuring absorbance at 570 nm [10,27,65].

An ANOVA test (single-factor analysis of variance) was used for statistical analysis to evaluate the significant difference in viability between the samples and the control samples (i.e., ADMEM + 5 wt.% FBS). A probability of less than 0.05 (*p*-value < 0.05) was considered statistically significant.

## 3. Results and Discussion

### 3.1. Material Preparation

To develop an advanced wound dressing material that can be used to treat painful wounds, we incorporated a non-steroidal analgesic (i.e., DCF) into a highly hydrophilic methacrylate-based polymer blend. SMNPs (i.e., FePt NPs) were included as an additive to control the release of DCF and to enable tailoring of the other dressing properties (as discussed in detail below). Both methacrylate-based polymers (i.e., PHEMA and PHPMA) exhibit good biocompatibility and contribute to the high hydrophilicity of the dressing. They have high thermal stability, are acid- and alkaline-hydrolysis-resistant, and possess tunable mechanical properties. Moreover, the slow degradation rate makes them suitable for use in long-term medical applications. Therefore, they are particularly attractive materials for drug delivery systems [70,71,72].

In this study, dose adjustment was achieved by incorporating DCF into multiple layers. For this purpose, we used an LbL approach that allows the fabrication of well-defined thin films, easy manipulation of the preparation conditions, and the fabrication of a wide range of materials [73,74,75]. Within this study, we have optimised some previously published approaches for multilayer film preparation [7,10,27,33,59,66].

To our knowledge, a multilayer analgesic dressing with two active compounds (DCF and FePt NPs) incorporated into a polymer composite (PHEMA/PHPMA) has not previously been reported. Methacrylate-based matrices for the controlled release of DCF have been reported in the literature, including EUDRAGIT^®^ polymers (Evonik Operations GmbH, Essen, Germany) [76,77,78]. Among our research questions was also to determine how variation in a drug-loaded nanolayer’s physicochemical properties (e.g., layer thickness, porosity) could be used to tune the release time and rate. The preparation of the spin-coating formulation required several optimisation steps to achieve the desired thickness of multilayer film and reproducibility. As mentioned earlier, the LbL spin-coating process provides the ability to control the layer thickness with high precision and accuracy by adjusting some parameters, including the concentration and viscosity of the solution, spin speed, and spin time. When the solution is prepared, a higher concentration and/or viscosity of the spin-coating solution usually results in thicker layers. In terms of technical aspects, adjusting the spin-coating process’s speed and duration can also impact film thickness. Thinner coatings can be obtained with higher spin speeds or with shorter spin times, while thicker films generally require lower speeds or longer durations [73,74,75]. The similar composition of the commercially available Altrazeal™ transforming powder dressing (TPD, Uluru Inc., Addison, TX, USA) [79] and our previous work on drug-containing coatings [10,27,33,66,80] provided a solid basis for testing the different concentrations and ratios of the polymers used, which were then optimised in terms of solution viscosity for the final spin-coating solutions. Since they are interrelated, the operating parameters (e.g., spin speed, spin time) and the volume of solution applied were gradually optimised to achieve the desired thickness and roughness. Moreover, the total thickness of the multilayer films also depends on the number of layers. As we have shown previously, the total thickness of the multilayer film increases linearly with the number of layers applied [33]. After optimising the key parameters, the prepared multilayer dressings resulted in a visually “smooth” and reflective surface. The optimised types of dressings used in further work are summarised in Table 2.

### 3.2. Characterisation

#### 3.2.1. Hydrophilicity Evaluation through Water Contact Angle Measurement

One of the fundamental principles of wound treatment is to keep the wound surface moist. An open wound dries out when exposed to air, resulting in scabbing or scarring. The latter is an important mechanical barrier that impedes epidermal cell migration and slows wound healing [80,81]. In addition, sufficient hydrophilicity is required for the administration of topical formulations to reduce potential discomfort to the patient and avoid potential irritation, particularly in very dry skin [82]. Therefore, we evaluated the hydrophilicity of prepared materials by the static contact angle measurements; the corresponding results are presented in Table 3. The contact angle measurement is usually considered the primary method to indicate the degree of wetting of similar formulations [10,33,83]—the smaller the contact angle, the better the wetting of the surface [84,85].

From the obtained values, we can conclude that all formulations are very hydrophilic, as indicated by angles well below 90° [10,86]. For the “base material” (D1), this was to be expected as both methacrylates used (e.g., PHEMA, PHPMA) are renowned for their hydrophilic nature due to the hydroxy, -OH, groups of the monomers [25,87,88]. As emphasised above, the hydrophilic nature of the wound dressing material is beneficial for successful skin regeneration and can also improve drug release efficiency. Highly wettable surfaces can also act as a “pumping system” that absorbs excess biological fluids from the wound. Moreover, contact between the hydrophilic surface and wound exudate allows the dissolution of drugs and/or diffusion from the materials into the wound bed. A hydrophilic character of the material is also advantageous for topical administration because it allows passive targeting strategies for treatment [10,89,90,91].

Incorporated FePt NPs slightly contributed to the improved wettability of the multilayer nanocomposite dressings (i.e., D3 and D4). Several factors regulate the refinement of properties in nanocomposites when NPs are incorporated into a polymer matrix. Coupling hydrogels with NPs can improve the mechanical properties of the former and impart new properties such as magnetic and optical properties and stimuli-responsive ability [92]. The unique combination of the hydrogel’s intrinsic viscosity and the stiffness of the NPs enables the tailoring of the physicochemical features of hydrogels [62,92]. Based on the literature, we hypothesised that the enhanced hydrophilicity/wettability of the nanocomposite-based dressings arises from the large interfacial region formed at the polymer-nanoparticle interface [92,93].

Although DCF is generally considered a hydrophobic drug [56], it did not significantly affect the overall hydrophilicity of the two DCF-containing multilayer dressings (i.e., D2 and D4). The surface contact angles observed for samples D2 and D4 increased by only 13.5° and 11.04°, respectively, indicating that the samples retained their hydrophilic character.

DCF has an amphiphilic character (with a hydrophilic carboxylate group and a hydrophobic part in the structure). The high hydrophilic character of the D2 and D4 samples indicates that the binding of this drug in the polymer mixture is mainly through hydrophobic interactions [72]. In the case of the nanocomposite sample (D4), the higher wettability is most likely due to the very small dimensions of the FePt NPs, which led to an increased surface volume ratio and larger surface areas for effective interactions with water to occur [92,93,94]. According to previous studies, the increased hydrophilicity of the samples could also be related to the surface roughness of the samples, where the formation of pores and sites for liquid penetration leads to a lower contact angle [95,96]. The material’s initial hydrophilicity (and hence its wetting capability) can significantly affect the kinetics of drug release. The absorption sites of the base material become saturated with water molecules, resulting in altered porosity and mechanical strength. In addition, the material’s moisture content also influences the mechanical behaviour of the stratum corneum by counteracting the effects of water loss [83,97,98,99].

Conversely, changes toward more hydrophobic values could potentially limit the therapeutical efficiency of incorporated drug molecules [10]. The slightly more hydrophobic character of the two DCF-containing multilayer dressings reduces the penetration of the incorporated drug, thus allowing a slower drug release. Based on the results, we can conclude that as-prepared multilayer dressings, regardless of the composition, have sufficient hydrophilicity to be considered potential drug-releasing formulations for wound care applications.

#### 3.2.2. Structural Properties by AFT-IR Spectroscopy

**Figure 2:**
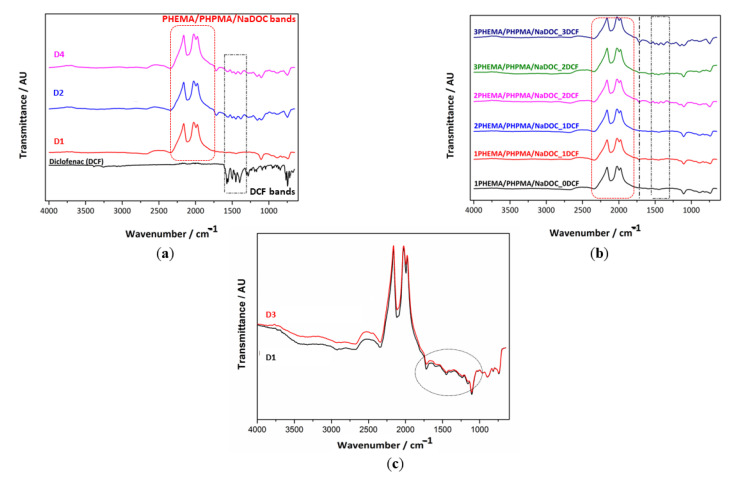
ATR-IR spectra of different multilayer samples: (**a**) proof of DCF incorporation, (**b**) presentation of the alternating layer applications (presented from the bottom up), and (**c**) investigation of the potential influence of incorporated FePt NPs polymer chemical structure.

In this work, we aimed to generate novel multilayer nanocomposites that form the basis for establishing an analgesic wound dressing. As shown below, the latter can be accomplished by combining two polymers, an anti-inflammatory drug DCF, and superparamagnetic FePt NPs. The multilayer structure of the material offers the possibility of fine-tuning the dosage (additional layers increase the dose) and release kinetics of the drug (e.g., through additional non-DCF-containing layers and the added FePt NPs). The ATR-IR analysis (Figure 2) was used to confirm the effective entrapment of both active ingredients (DCF molecules and superparamagnetic FePt NPs) (Figure 2a) and the multilayer character of the prepared coatings (Figure 2b).

The incorporation of DCF layers resulted in a shouldered band appearing in the region between 1750 and 1250 cm^−1^ (indicated by a dotted black rectangle in Figure 2a). The characteristic bands for DCF are related to (1) the intense stretching of the aromatic ring at 1603 and 1556 cm^−1^, (2) a band at 1507 and 1500 cm^−1^ associated with the C-N-H bending of the secondary amine and the C-H vibration of the aromatic rings, (3) a band at 1468 cm^−1^ attributed to the C-N stretching and the C-H vibration (aromatic ring), and (4) bands at 1452 and 1305 cm^−1^ attributed to the methyl (-CH_2_) bending [100,101,102]. In addition, the bands around 1580–1584 cm^−1^ are likely due to -C=O stretching vibrations. The lower intensity of these bands in the DCF-containing samples (compared to the pure DCF) indicates the occurrence of electrostatic interactions between DCF and the polymer (nano)composites [10,56,59,65,102]. The shift of some characteristic DCF bands from their original position in the multilayer (nano)composites potentially indicates the involvement of hydrogen bonds between the compounds [56].

The alternating appearance and absence of characteristic bands associated with pure DCF (areas marked in black in Figure 2b), a typical feature of multilayer dressings, confirms the multilayer character of the D2 and D4 samples. Their intensity is more pronounced after the second F1 layer (from the blue spectra) and increases with the next DCF layer. The most distinct and well-defined peaks in the range from 2300–1750 cm^−1^ (marked red areas) could be related to the base material (i.e., D1) and correspond to the stretching vibration of the methylene groups (-CH_2_) at 2250 cm^−1^ and the stretching of the ester bonds of the methacrylic and ethyl acrylic groups between 2100 and 1750 cm^−1^ [48,103]. The position of the vibrational peaks of the carbonyl groups (C=O) at about 1750 cm^−1^ indicates the formation of intermolecular hydrogen bonds in the structure of D1 [104]. Regardless of the actual nature of the vibrations, the visible increase in the intensity of typical peaks with the subsequent layers suggests that an LbL structure was successfully generated using the spin coating technique. Additionally, these measurements confirmed the effective inclusion of DCF into the multilayer dressing [59,65,66]. The reasons for including FePt NPs in the formulation were the expected positive influence on the mechanical and physicochemical properties [62], which in turn could enable tailoring of the release kinetics. Considering our previous results and other literature sources, even an effective targeting of the analgesic to the injury site to achieve a high therapeutic effect could be expected [20,105]. Finally, the facilitation of the transport of hydrophobic molecules through the stratum corneum [41] was also reported in the literature, which might further boost the effect of the proposed dressing.

Since NPs generally act as fillers and affect the physicochemical properties of the material, we evaluated the possible effects of incorporated FePt NPs on the chemical composition of the base polymer matrix (i.e., D1). For this reason, we prepared two drug-free samples (D2 and D4, respectively). The corresponding spectra (see Figure 2c) contained all the characteristic bands of the polymer matrix (D1): the -CH_2_ bending mode at 2250 cm^−1^ and the stretching vibration of the C=O between 2100 and 1750 cm^−1^. Careful examination reveals a minimal shift of the bands in the nanocomposite, 1750 and 1200 cm^−1^ region (dotted line in Figure 2c), to lower wavelengths that could contribute to the stretching vibrations of the Fe-O bond due to the vibrations of the metal ion-oxygen (M-O) complex [106]. However, this is negligible as a marker for distinguishing FePt NPs in polymer-based multilayer coatings. Therefore, we can assume that the in situ incorporation of FePt NPs did not alter the chemical structure of the base polymer matrix (i.e., D1). In addition, the characteristic DCF bands present in D2 and D4 (see Figure 2a) indicate drug loading. Altogether, these results confirm that the incorporated FePt NPs do not affect the individual properties of the polymer or the drug [107].

#### 3.2.3. Sample Morphology and Roughness

**Figure 3:**
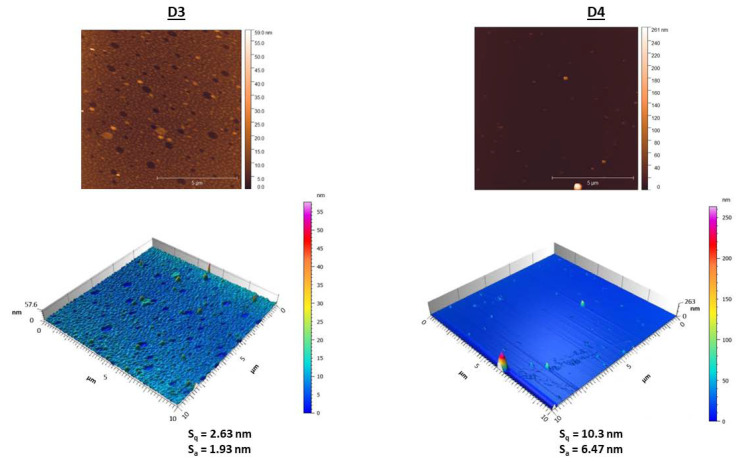
Surface topography and roughness parameters measured by AFM at the surface of nanocomposites (D3 and D4).

Surface topography and chemistry can affect the physicochemical properties of a material, including its hydrophilic behaviour and adhesive tension [108,109]. AFM was used to study the influence of the incorporated DCF and FePt NPs on the surface properties of the prepared samples. The changes in nano-topographic features of the nanocomposites (D3 and D4) were especially in focus. For a better illustration and a clearer visualisation, only the measurements of the top layer of the multilayer nanocomposites are shown in Figure 3. Even after briefly comparing the obtained images, important conclusions can be drawn. First, the incorporated DCF seems to affect the surface features/patterns, indicating that the surface of the substrates is covered differently over the entire available area compared to the DCF-less samples. Consequently, this significantly alters the substrate morphology (and key features), which might also affect the release kinetics (see further details below). Second, both nanocomposite surfaces have a similar roughness, while the nanocomposite with the additional DCF (i.e., F2) layer has a slightly rougher surface. The increase in roughness is likely due to a change in molecular organisation in the polymer matrix due to the incorporation of the FePt NPs and DCF, resulting in different surface patterns. For the drug-free nanocomposite (D3), the addition of FePt NPs changed the surface height distribution by affecting the stretching of the polymer chains near the surface of the NPs. As the FePt NPs act as fillers on or in the polymer surface, the interaction at the polymer-metal interface strengthens the adhesion between the polymer chains and the FePt NPs, which contributes to the smoothness of the surface, which might explain the changes in the height distribution [110,111]. The visible morphological differences in the nanocomposites can also be associated with the semicrystalline form of the DCF molecule. The higher density of potential electrostatic forces formed between F3 and DCF, which might contribute to increased system viscosity, is still pronounced when covered with another amorphous F3 layer. The strength of these electrostatic forces impedes the diffusion of DCF and allows crystals to grow [112], which is reflected in sharper surface edges (a common feature of crystalline materials). Thus, this indicates that the DCF in D4 is partially recrystallised [59,113]. In general, the LbL spin coating process proved to be a viable method for preparing homogeneous surface films regardless of the multilayer coating composition, as only minor differences in surface morphology were observed between the individual samples.

Since AFM analysis is considered the most powerful technique for characterising heterogeneous systems on the nanoscale, we have also used it to verify the presence of FePt NPs in nanocomposites [114]. The topographic images (Figure 3) show the embedding of the FePt NPs in the polymer matrix. In order to approximate the size of the visible NPs, the measurements were performed in different areas of each nanocomposite. For biomedical applications, the optimal nanoparticle size is in the range of 10–50 nm [50,105,106], including the FePt NPs used in this study.

### 3.3. Functional Testing

#### 3.3.1. In Vitro Drug Release Testing

**Figure 4:**
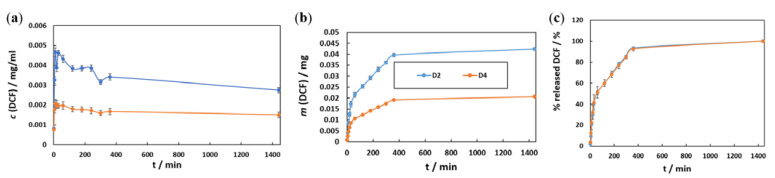
The in vitro drug release in PBS solution at 37 °C: (**a**) the DCF mass concentration (aliquot take-off with subsequent PBS dilution needs to be taken into account, which is why the concentration drops) and (**b**) the cumulative released mass of DCF, while (**c**) shows the percentage of released DCF as a function of time.

Appropriate wound care is essential to prevent infection, promote skin regeneration, and manage wound-associated pain [115]. To achieve the latter, controlled and local release of NSAIDs with a prolonged analgesic (and anti-inflammatory) effect is an important feature to be considered in contemporary wound dressings. Understanding the latter is essential, as concentration fluctuations may represent the difference between therapeutic (desired) effects and local toxicity [59,116]. One of the most important features of a drug delivery system is to ensure that the amount of drug released is within the therapeutic window. Therefore, we studied drug release mechanisms, and the obtained DCF release profiles (Figure 4) are interpreted in terms of released DCF concentration, cumulative released DCF mass, and percentage of DCF released as a function of time.

The changes in DCF concentrations as a function of release time during the in vitro release assay are presented in Figure 4a. The multilayer structure of the D2 and D4 samples with alternating sequences of drug and polymer layers (with or without FePt NPs) (as illustrated in Figure 1) is reflected in a characteristic pulsatile release profile during the first 360 min of release [10,27,59,65,66]. In general, drug release mechanisms from polymer carriers include diffusion, dissolution, desorption of the drug, osmosis, swelling, and/or polymer degradation, each of which can result in a specific pattern of drug release [117]. In the case of hydrogel matrices such as PHEMA and PHPMA, the drug release is regulated mostly by diffusion and matrix swelling [27,75,118]. Since DCF is soluble in polar solvents (e.g., ethanol and water), the faster the diffusion of buffer solution in F1, the faster the drug release is expected [118]. In addition, the observed alternating curve shape (i.e., three peaks and three valleys) corresponds to the actual number of DCF-containing and DCF-free layers. This mechanism of DCF release from (nano)composites is known as burst release [27,59] and is characterised by a rapid release of the drug upon direct contact with the aqueous medium. Small amounts of released DCF from the underlying DCF layers also contribute to an overall rapid release pattern in the initial part of the curve. The alternating burst effects are followed by sustained release due to the DCF-free layers acting as a barrier to the mass transport of DCF [27,56,59,65]. The diffusion of DCF from the multilayer coating is driven by the swelling behaviour of the polymer matrix [119]. Due to its hydrophilic nature, the water molecules should cause relaxation and/or deformation of the polymer chains, which promotes DCF release [120]. Therefore, the degradation of the polymer matrix by water molecules when exposed to the medium can lead to its partial degradation or even detachment, exposing DCF molecules [27,59,65]. This burst and sustained release pattern repeat until the total amount of DCF incorporated is depleted, accompanied by the dissolution of the DCF layers.

Considering the potential clinical application of the developed wound dressing material, we can predict that it would swell due to wound exudate and blood exposure, leading to an increased drug release rate. Therefore, additional drug-free layers between the DCF-containing layers as a barrier to the DCF might be beneficial to ensure prolonged drug release [27,56,59,65]. The incorporated FePt NPs did not significantly affect the release mechanism per se; however, their possible influence could be seen in the cumulative dose of DCF in a given time. Presumably, the formation of strong duplex interactions led to increased repulsive forces, which subsequently prolonged the durability of DCF in nanocomposites [10,121]. Additionally, the temperature of the medium (e.g., 37 °C) did not result in increased drug release from the D4 sample. Magnetic NPs, including FePt NPs, are susceptible to several external stimuli, such as pH and temperature. The localised heating near NPs elevates temperatures at the magnetic surface of NPs, which can accelerate the hydrolysis reaction rate of the polymer matrix [122]. Thus, under physiological conditions (i.e., at pH 7.4 and 37 °C), the incorporated FePt NPs did not affect the stability of the chemical bond between DCF and polymer matrix (consistent with the results of the AFT-IR analysis), resulting in slow hydrolysis kinetics. In this case, the amount of released DCF from the material is mainly determined by the concentration gradient between DCF-containing and DCF-free nanocomposite regions.

The effects of superparamagnetic FePt NPs on drug release mechanisms are partly related to the size of the NPs. Due to a larger surface-to-volume ratio, smaller NPs allow for faster drug release, while larger NPs require more time to diffuse the drug out of the nanocomposites [123,124].

The values of the cumulative release of DCF as a function of time were plotted in Figure 4b. The results show significant differences in the mass of DCF released from both systems. It seems that DCF release is favoured by “pure” polymer (i.e., F1) layers, which is expected given the earlier results (in Figure 4a). In the case of the multilayer nanocomposite system, two possibilities could shed light on a poorer release yield. First (as mentioned above in Section 3.3.1), due to enhanced interaction between the DCF and the FePt NPs. As nanomaterials, the FePt NPs have their own physical/chemical properties, most likely determined by the properties of the SiO_2_ substrate [125]. Second, the FePt NPs could affect the actual layering during the spin-coating process. The addition of FePt NPs to the polymer matrix not only induces the property of superparamagnetism [121], which is not in this study’s focus but also affects the hydrophilicity, which could lead to altered contact between F1 and F2 layers. Moreover, the potential “complexation interactions” between the NPs and F1 could cause a different packing of the molecules in the polymer matrix, leading to a significant decrease in the size and concentration of the H-bonded clusters in the aromatic (phenyl) ring of the methacrylate-based polymers (i.e., PHEMA and PHPMA). Consequently, this disorder in the hydrogen-bonding structure [126] could lead to lower interactions with DCF molecules. Despite the different release concentrations, both samples exhibit the same release mechanism, as evidenced by the similar shape of the curves. The whole-time release profile can be divided into three distinct phases. Phase I had a short duration with a rapid drug release rate (in the first 30 min), whereas phase II had a longer duration (up to 360 min) but with a slower release rate during which the DCF is released continuously. In phase III (i.e., release plateau), DCF is released at a slow rate from the remaining (undissolved) polymer layers (from 360 min to the end point of release). Based on these results, we can conclude that the LbL spin coating process offers a straightforward fabrication approach where the final DCF dose can be tailored by the number of coatings [66,116].

The mass fraction of DCF released as a function of time (Figure 4c) supports the almost identical release mechanisms mentioned above for both samples. In the first 30 min, about 40% of the DCF is released, regardless of the number of DCF layers and FePt NPs incorporated into the polymer matrix. Throughout 30 and 360 min, the mass fraction of the released drug increases to 96%. These results indicate the potential use of such DCF-containing formulations in medicine. The incorporated dose can be tailored to specific needs by simply adjusting the number of DCF layers in the respective multilayer coatings [59]. In addition, the penetration of DCF through the stratum corneum will be facilitated by added nanocomposites [41]. By modifying the content of the monomers in the polymer matrix, accelerated dissociation of the polymer matrix can be achieved by changing the phase transition temperature of the nanocomposite, leading to the release of DCF from the multilayer dressing [47,122]. Furthermore, these results, in conjunction with the different cumulative doses of DCF released from each sample, demonstrate the potential of these multilayer nanocomposite dressings to provide identical, dose-independent treatment, which can be adjusted to the patient’s needs. Adjusting the number of total layers of the dressing affects not only the therapeutic ability of the dressing but also its mechanical strength; increasing the number of alternative layers can improve the durability of the dressing. In addition, the incorporated FePt NPs as reinforcing fillers also contribute to the improved mechanical properties of the PHEMA and PHPMA matrices by increasing the density and reducing the size of the defects in the polymer matrix [127,128]. As such, they are promising candidates for long-term localised drug delivery systems for patient-specific therapy.

#### 3.3.2. Biocompatibility Testing Using Human-Derived Skin Fibroblasts

**Figure 5:**
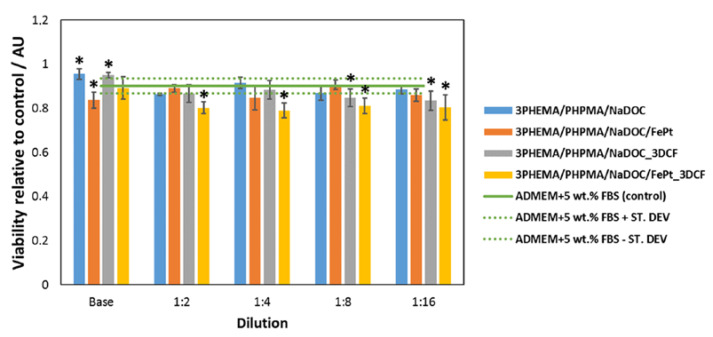
Biocompatibility test on skin fibroblasts (extract exposure method of MTT assay). The viabilities are presented as average relative viabilities compared to the control. Marked samples (*) showed a significant difference compared to the control sample (*p* values of <0.05).

Establishing appropriate biocompatible material properties is critical for any material’s safe use in biomedical applications. Therefore, we investigated the potential cytotoxic effect of multilayer coatings by biocompatibility testing using the extract exposure method of the MTT assay on human-derived skin cells. The MTT assay is a quantitative method routinely used to determine adherent cells’ mitochondrial function and cell viability. It is commonly used as a preliminary assessment of biomaterial toxicity according to the ISO 10993-5 guideline [129]. This regulatory norm recommends three types of cytotoxicity testing (extract dilution exposure and direct and indirect contact tests). The extract dilution exposure method is commonly applied to detect toxins leached from exposed materials [130]. An important consideration in selecting agents used in cytotoxicity testing is the cells and tissues likely to be affected by the tested substance. The multilayer dressings developed in this study are intended as dressing materials for skin distortions (e.g., for pain-relieving postoperative care) and, as such, come into contact with skin cells, including skin fibroblasts. Determining the viability of cells after exposure to specific materials has important implications for their safety and efficacy. It may point to the potential formation of toxic degradation products or extensive drug release, which could have local toxic effects (visible by inhibited cell proliferation and reduced cell viability) [59,130]. The pure cell growth medium (ADMEM + 5 wt.% FBS) was used as a comparative control in this extract exposure method. The potential cytotoxicity of the extracts of the prepared materials (at the original concentrations and their dilutions) on skin fibroblasts is shown in Figure 5. It is immediately apparent that the base polymer formulation (e.g., D1) performs better than the control sample (i.e., favours cell proliferation). The DCF-containing dressings (i.e., D2 and D4) also have a positive effect on cell viability. The improved cell viability of the DCF-containing (i.e., D2 and D4) samples compared to the drug-free (i.e., D1 and D3) samples is likely due to the anti-inflammatory properties of the DCF molecule [80,131,132]. Conversely, both samples with incorporated FePt NPs show a slightly negative effect on the cells. This could be due to the ability of the NPs, which were released into the cell growth medium after the dissolution of the polymer, to form reactive oxygen species (ROS). Elevated ROS levels cause significant damage to the DNA of the cells, leading to cell cycle arrest and subsequent cell death [133]. Nevertheless, all formulations were found to be safe, as none of the samples resulted in toxic effects (all values are well above 50% [134,135,136]) on the skin fibroblasts used, which was also confirmed by optical micrographs of the exposed skin fibroblasts.

Considering these results, the proposed multilayer dressings have a high potential for further testing to develop novel pain-relieving wound dressings.

## 4. Conclusions

This study demonstrates the design of a new multilayer dressing based on the simultaneous integration of magnetic NPs and the non-steroid anti-inflammatory drug DCF in a polymer matrix for wound care. In a series of testing methods, we have shown that the proposed wound dressing composition yields the desired properties for a long-lasting analgesic effect. The wound dressing could regulate the amount of drug released and limit the unwanted side effects. Moreover, including superparamagnetic FePts NPs could be beneficial in several aspects. They can effectively retain drug molecules in nanocomposite-based layers and improve penetration through the skin, as well as have a positive effect on the mechanical properties of the as-prepared wound dressing. Finally, we have demonstrated the safety of the proposed wound dressing on human skin fibroblasts. Overall, the proposed multilayer nanocomposite-based system could be a promising postoperative wound dressing to alleviate patients’ pain and discomfort.

## Figures and Tables

**Table 1:** Final compositions of prepared solutions.

	PHEMA	PHPMA	NaDOC	DCF	FePt NPs
	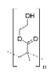	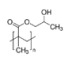	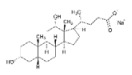	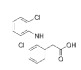	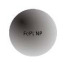
FORMULATION 1 (F1)	1	0.1	1		
FORMULATION 2 (F2)	1	0.1	1	1	
FORMULATION 3 (F3)	1	0.1	1		1
FORMULATION 4 (F4)	1	0.1	1	1	1

**Table 2:** Different types of dressings.

	Preparation
DRESSING 1 (D1)	3 layers of F1
DRESSING 2 (D2)	3 layers of F1 with alternating 3 layers of F2
DRESSING 3 (D3)	3 layers of F3
DRESSING 4 (D4)	3 layers of F3 with alternating 3 layers of F4

**Table 3:** Assessment of hydrophilicity by contact angle measurement.

Sample	Average Contact Angle Value (°)	Standard Deviation (°)	Photographs
D1	25.53	1.51	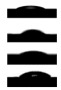
D2	39.03	0.64	
D3	21.89	1.18	
D4	32.93	1.31	

## Data Availability

All related data is part of the manuscript.
